# Production of an Animal Model of Semi-Yin and Semi-Yang Syndrome with Diabetic Ulcers and Study of Its Pathological and Metabolic Features

**DOI:** 10.1155/2021/6345147

**Published:** 2021-06-28

**Authors:** Yu Liu, Jian-Ping Shi, Wu Xiong, Yang Liu, Yu Yan, Chao-Qi Yin, Yu-Qi Jiao, Xi Zhang, Jian-Da Zhou

**Affiliations:** ^1^Postdoctoral Research Station of Clinical Medicine & Department of Plastic Surgery, The Third Xiangya Hospital, Central South University, Changsha 410013, China; ^2^Inner Mongolia Medical University, Hohhot 010000, China; ^3^The First Affiliated Hospital of Hunan University of Chinese Medicine, Changsha 410007, China; ^4^Department of Plast Surg, The Third Xiangya Hospital, Central South University, Changsha 410013, China; ^5^Clinical Medical College of Hunan University of Chinese Medicine, Changsha 410007, China

## Abstract

**Background:**

To create an animal model for diabetic ulcers with semi-Yin and semi-Yang (SYSY) syndrome and to study the pathological and metabolic features of SYSY syndrome.

**Methods:**

Firstly, based on the clinical characteristics of the SYSY syndrome of diabetic ulcer, an animal model of diabetic ulcers with SYSY syndrome being full-thickness skin defects was created by injecting streptozotocin (STZ) intraperitoneally, infecting with *Staphylococcus aureus*, and gastrically administering senna. Secondly, the content and distribution patterns of collagen fibers, the expression of neutrophils and macrophage markers, angiogenesis, and the expression of IL-1*β* and IL-10 in the rats with Yang syndrome, Yin syndrome, and SYSY syndrome of diabetic ulcers at different time points were detected. Representative traditional Chinese medicine (TCM) ointment of Yang syndrome, Yin syndrome, and SYSY syndrome was used to treat this animal model. The above indexes in each treatment group were detected. Finally, metabonomics was used to detect and analyze the changes of differential metabolites related to macrophage metabolism in Yang, Yin, and SYSY syndromes at different time points.

**Results:**

An animal model of diabetic ulcers with SYSY syndrome was established. The pathological features of the SYSY syndrome group were chronic low-grade inflammatory reactions. On the third day, the SYSY syndrome group displayed lower expression of CD16, CD68, CD163, IL-1*β*, and metabolites related to M1-type macrophages compared with other groups. On the seventh day, the SYSY syndrome group showed lower expression of CD31, IL-10, myeloperoxidase, and metabolites related to M2-type macrophages. Treatment with Chong He Ointment, a representative TCM ointment for SYSY syndrome, reversed the expression levels of these indexes and promoted wound healing in the SYSY group.

**Conclusion:**

SYSY syndrome presents a persistent pathological state of low inflammation, which may be caused by an insufficient activation of the M1-type metabolic pathway in macrophages in the early acute inflammatory stage, resulting in the incomplete clearance of pathogens and debris and continuous stimulation of macrophages to initiate the M1-type metabolic pathway. CD163, CD31, IL-10, and citric acid can be used as potential specific markers for the recovery and progression of SYSY syndrome.

## 1. Introduction

Globally, 422 million people have been diagnosed with diabetes, of which, approximately 15% develop leg ulcers [1]. Early treatment is the key to reducing the amputation rate in diabetic foot patients [2].

Many traditional Chinese medicine (TCM) ointments effectively treat diabetic ulcers, and they show a more significant curative effect during the early stages of treatment than in the later stages [3]. However, most Chinese herbal preparations for external application show intended results only after the correct differentiation of TCM syndrome.

TCM syndrome refers to an abstract collection of pathological changes at a certain stage in the clinical course of the disease that thoroughly reveals the cause of the disease. Different syndromes have different metabolic characteristics and susceptibilities [4]. Diabetic ulcers fall into the category of “sore and ulcer” in TCM [5]. TCM science categorizes “sore and ulcer” into the following syndromes: Yang syndrome (wounds show signs and symptoms of redness, swelling, heat, and pain), Yin syndrome (dull skin, stasis, coldness, and numbness), and semi-Yin and semi-Yang (SYSY) syndrome (mild or no redness, mild or no swelling, mild or no heat, and mild or no pain) [6]. SYSY syndrome refers to the stage at which Yang syndrome turns into Yin syndrome, and it is the most difficult TCM syndrome to differentiate. Timely and accurate identification of this syndrome plays a significant role in preventing “sores and ulcers” from Yang syndrome to develop into refractory Yin syndrome. However, the subjectivity and abstractness in the differentiation of TCM syndromes lead to different diagnostic results for the same diabetic ulcers. Different diagnoses of TCM syndromes lead to the selection of different TCM ointments for treatment, and the final therapeutic effect is also different. Therefore, indicators for diabetic ulcers in SYSY syndrome should be developed scientifically and objectively to provide a laboratory basis to modernize TCM treatment and make it more targeted.

Animal models are important for TCM syndrome research, but no animal model has been developed for SYSY syndrome till now. Therefore, we conducted this study to develop an animal model for diabetic ulcers in SYSY syndrome based on the clinical characteristics of diabetic ulcers in this syndrome. We studied the pathological and metabolic characteristics of SYSY syndrome and searched for biological targets, so as to provide evidence-based medicine for TCM syndrome and provide an objective theoretical basis for guiding clinical medication based on syndrome differentiation.

## 2. Methods

### 2.1. Reagents

Streptozotocin (STZ, S0130-1G, Sigma), methanol (67-56-1, HPLC-grade, CNW Technologies), chloroform (67-66-3, HPLC-grade, Adamas), pyridine (110-86-1, HPLC-grade, Adamas), methoxyamine hydrochloride (593-56-6 AR, TCI Chemicals), adonitol (488-81-3 ≥ 99%, Sigma), BSTFA with 1% TMCS(Regis Technologies), fatty acid methyl esters (FAMEs, Dr. Ehrenstorfer), hematoxylin stain (G1004-500 ML, Servicebio), eosin dye solution (G1001-500 ML, Servicebio), Masson's trichrome stain (G1006-100 ML, Servicebio), anti-CD68 (GB11067, Servicebio), antimyeloperoxidase (MPO, GB12224, Servicebio), anti-CD31 antibody (ab182981, Abcam), anti-CD16 antibody (ab198507, Abcam), anti-CD163 antibody (ab182422, Abcam), IL-1*β* (RA20020, Bioswamp), IL-10 (RA20090, Bioswamp), VEGF (A5609, Affinity), and VEGFR1 (A17877, ABclonal) were used in this study.

### 2.2. Traditional Chinese Medicine

According to the Orthodox Manual of External Diseases, Chong He Ointment (CHO) is composed of *Cortex Kadsurae Radicis, Angelicae Pubescentis Radix, Radix Paeoniae Rubra, Acori Graminei Rhizoma, and Radix Angelica* [6]. These herbs were crushed after inspection, filtered through a 40-mesh sieve, and mixed well. Following this, 50 g of powder was weighed each time, and it was added to 400 mL double-distilled water. Subsequently, the heat-reflux-extraction process was performed for 1 h, and the filtrate was collected using gauze. Next, for extraction, 400 mL of 95% ethanol was added to the filtrate for 1 h. The two filtrates were mixed, frozen, and dried to obtain a freeze-dried powder. Lanum was used to make the ointment: Jin-huang Ointment (JHO, Approval Number: 1609192, Longhua Hospital Affiliated to Shanghai University of Traditional Chinese Medicine); Hui-yang Yu-long Ointment (HYYLO, Huibentang Biological Technology Co., Ltd.); Erythromycin Ointment (EO, approval number: National Pharmaceutical Approval H41020138, Xinxiang Huaqing Pharmaceutical Co., Ltd.); *senna* leaf granules (National medicine approval number: Z10910006, Jiangsu Eddie Pharmaceutical Co., Ltd).

### 2.3. Bacterial Strain

*Staphylococcus aureus* was purchased from the China Institute for Food and Drug Control (CMCC26112-10).

### 2.4. Instruments

Gas chromatography (GC-2030 Shimadzu Corporation), mass spectrometer (QP2020NX Shimadzu Corporation), chromatographic column (DB-5 ms, Agilent), centrifugation machine (Heraeus Fresco 17, Thermo Fisher Scientific), electrophoresis apparatus (EPS-300, Tanon), enzyme standard instrument (muLISKANMK3, Thermo Fisher Scientific), rotary type slicer (RM2255, Leica Microscopy System Co., Ltd.), and paraffin embedding machine (Leica Microscopy Systems Co., Ltd.) were used in the study.

### 2.5. Animals

Male Wistar rats (2 months old, 180–220 g, SPF grade) were provided by the Animal Experimental Center of Inner Mongolia Medical University (SCXK 2020–0001). The rats were housed in animal laboratories under 22–24°C and 40%–60% relative humidity, and they were provided standard laboratory feed and water. The study was approved by the Evaluation Committee of Inner Mongolia Medical University, and it was conducted in accordance with the regulations of the State Administration of Animal Laboratory Animals.

### 2.6. Diabetic Ulcer Rats with SYSY Syndrome

Rats were intraperitoneally injected with 50 mg/kg STZ for 15 days. Rats with symptoms of overeating, overdrinking, and urorrhagia were induced to develop diabetes. The model was successfully created when the blood glucose levels for at least three random tests were found to be more than 16.7 mmol/L [7]. The diabetic rats were depilated in the middle and upper parts of the lumbar spine after anesthesia. A perforator was used to create a 2 cm × 2 cm deep round wound into the fascia [8].

A total of 2 mL of *Staphylococcus aureus* suspension (1.5 × 10^9^ cfu/mL) was applied to the wound surface of diabetic rats with full-thickness skin defects. The Yang syndrome model was created when acute inflammatory reactions, such as redness and swelling, were observed [9, 10].

Forty rats with Yang syndrome were randomly divided into four experimental groups (*n* = 10/group). *Senna* (0.6 g/d) was orally administered to each group for three days, 0.6 g/d, 0.9 g/d, and 0.9 g/d for 6 days, 3 days, and 3 days, respectively (the treatment dose of *senna* per day for each rat was 0.312 g/d, calculated according to the table of dose conversion coefficient between animals and humans).

According to the local and systemic symptoms of diabetic ulcer rats observed in the preexperiment data and TCM literature on symptoms of Yang syndrome, SYSY syndrome, and Yin syndrome, indicators were scientifically developed by independently creating a Quantitative Scale for TCM Syndromes in Diabetic Ulcer Rats (Table 1) [9, 11]. Systematic and local symptoms of rats in each group were observed and scored according to the Quantitative Scale. GraphPad Prism 7.0 was used to create a heat map with scoring results in each group, and the data were visualized (pink = Yang syndrome, purple = SYSY syndrome, and black = Yin syndrome). The data were used to identify the time point and dose at which diabetic ulcers changed from Yang syndrome to SYSY syndrome.

### 2.7. Pathological Characteristics and Biomarkers of the Animal Model of Semi-Yin and Semi-Yang Syndrome

**Figure 1:**
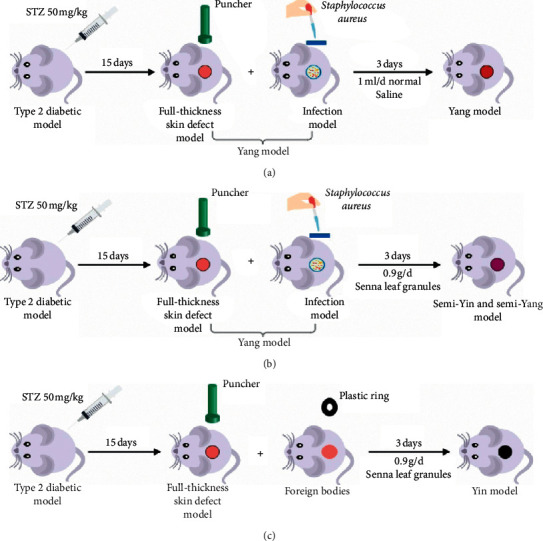
Diabetic ulcer rats with different traditional Chinese medicine (TCM) syndromes. (a) Yang syndrome model. (b) SYSY syndrome model. (c) Yin syndrome model.

Seventy-two diabetic rats with full-thickness skin defects were randomly divided into the SYSY syndrome treatment group (Figure 1(b)), Yang syndrome control group (Figure 1(a)), and Yin syndrome control group (Figure 1(c)) (*n* = 24/group). The Yang syndrome model was created as previously described. *Senna* (0.9 g/d) was orally administered to rats in the SYSY syndrome group for three days on the basis of the Yang syndrome model. Yin syndrome model was created by inserting a sterile plastic ring (inner diameter: 2 cm; outer diameter: 2.5 mm) into the wound edge of diabetic rats with full-thickness skin defects, and the wound was sutured and bound up with gauze. Following this, 0.9 g/d senna was orally administered for three days.

Local wound and systemic symptoms of rats in each group were observed daily to record the blood glucose levels and body weight. According to the Quantitative Scale for TCM Syndromes of Diabetic Ulcer Rats scoring results, the animal model was determined to be consistent with the clinical characteristics of SYSY syndrome.

After 3, 7, and 14 days, six rats in each group were randomly sacrificed, and the wound tissue (within 0.5 cm of wound edge) and serum were collected. Histopathological changes in wound healing were observed using hematoxylin and eosin (H&E) staining. Masson's trichrome staining was used to evaluate the formation of collagen fibers during wound healing. The expression of CD31, CD16, CD68, CD163, and myeloperoxidase (MPO) was detected using immunohistochemical staining, and the changes in vascular endothelial cells, macrophages, and neutrophils during the process of wound healing were evaluated. Serum expression levels of IL-1*β* and IL-10 during wound healing were detected using enzyme-linked immunosorbent assay (ELISA). Gas chromatography coupled with time-of-flight mass spectrometry (GC-TOF-MS) was used to detect the changes in differential metabolites during the wound healing process.

Testing of the reliability of the animal model of SYSY and efficacy of evaluation indicators.

One hundred and twenty rats with SYSY syndrome were randomly divided into a model group (SYSY) and four experimental groups (*n* = 24 rats/group). The wounds of the four experimental groups were treated with Chong-he Ointment (CHO), a Chinese medicine prescription for the treatment of SYSY syndrome, Jin-huang Ointment (JHO), a Chinese medicine prescription for the treatment of Yang syndrome, Hui-yang Yu-long Ointment (HYYLO), a Chinese medicine prescription for the treatment of Yin syndrome, and erythromycin ointment (EO) (2.6 g/kg) from 8 : 00 to 9 : 00 a.m. every day [12, 13].

Wound and systemic symptoms were observed and recorded. Changes in the TCM syndromes of rats in each group were evaluated according to the scoring results of the Quantitative Scale for TCM Syndromes in Diabetic Ulcer Rats.

Six rats in each group were randomly sacrificed, and the wound tissues and serum were collected after 3, 7, 14, and 21 days of an external application of ointments. H&E staining, Masson trichrome staining, immunohistochemical staining, ELISA, and GC-TOF-MS were used to detect the biological markers of SYSY syndrome.

### 2.8. Calculation of Wound Area Using NIH Image J1.48

The collected images were processed using NIH Image J1.48 to calculate the area of the wound. Two independent, uninformed observers used the image analysis software to measure the initial wound and wound contraction areas. The wound contraction area was calculated according to the following equation: wound contraction rate (%) = (wound contraction area [C])/(initial wound area [I]) × 100%.

### 2.9. Assessment of the Histopathology of Wounds Using H&E Staining

Wound tissues in 10% neutral buffered formalin (NBF) were subjected to H&E staining according to the manufacturer's instructions, and they were observed using an Image Scope camera.

### 2.10. Observation of the Expression Level of Collagen Fibers in Wound Tissues Using Masson's Trichrome Staining

The wound tissues in 10% NBF were subjected to Masson's trichrome staining according to the manufacturer's instructions, and they were observed using an Image Scope camera.

### 2.11. Observation of the Expression Level of Angiogenesis, Neutrophils, and Macrophages in Wound Tissues Using Immunohistochemical Staining of CD31, MPO, CD16, CD68, and CD163

The detection index of each wound tissue in 10% NBF was determined according to the manufacturer's instructions, and the tissues were observed using an Image Scope camera. Immunohistochemistry data were quantitatively analyzed using Image-Pro Plus 6.0, in which CD31 was detected using the vessel counting method. MPO, CD16, CD68, and CD163 were analyzed by IHC profiler plug-in. According to the formula Score=(Number of pixelsinazone) × (score of the zone)/Total number of pixelsin the image, the computer grades the results into high positive (+++), positive (++), low positive (+), and negative (−). All quantitative analysis methods were conducted under 200*x* field of vision, and they used the same brownish-yellow color as the unified criteria for positive judgment. The experiments were performed at least thrice for each photo.

### 2.12. Detection of IL-1*β* and IL-10 Levels in Rat Serum Using ELISA

The wound tissues were stored in a refrigerator at −80°C, and they were used for ELISA. IL-1*β* and IL-10 were measured using IL-1*β* and IL-10 kits, respectively, according to the manufacturer's instructions.

### 2.13. Detection of Metabolic Substances in Wound Tissues Using Gas Chromatography Coupled with Time-of-Flight Mass Spectrometry

The sample (25 mg) was weighed and 500 *μ*L of extract solution was added. The samples were then homogenized and sonicated for 5 min. The samples were then incubated at −40°C for 1 h, and they were centrifuged at 12000 rpm (RCF = 13800 × *g*, *R* = 8.6 cm). The supernatant (100 *μ*L) was transferred into EP tube. Following this, 20 *µ*L of each sample was mixed in QC samples, and dry extract and 5 *μ*L adonitol were added in a vacuum concentrator. Then, 30 *μ*L of methoxyamination hydrochloride (20 mg/mL in pyridine) was added, the sample was incubated at 80°C for 30 min, and it was derivatized using 40 *μ*L of BSTFA regent (1% TMCS, v/v) at 80°C for 1.5 h. Gradually, 5 *μ*L of FAME (in chloroform) was added to the QC samples. All samples were then analyzed using GC-TOF-MS.

GC-TOF-MS analysis was performed using a Shimadzu GC-2020 gas chromatograph coupled with a mass spectrometer. The system utilized a DB-5 ms capillary column. A 1 *μ*L aliquot of sample was injected in split (5 : 1) mode. Helium was used as the carrier gas, the front inlet purge flow was 3 mL min^−1^, and the gas flow rate through the column was 1 mL min^−1^. The initial temperature was maintained at 50°C for 1 min, raised to 310°C at a rate of 8°C min^−1^, and held at 11.5 min. The injection, transfer line, and ion source temperatures were 280°C, 280°C, and 200°C, respectively. The energy was −70 eV in the electron impact mode. The mass spectrometry data were acquired in full-scan mode with an m/*z* range of 50–500 after a solvent delay of 7.2 min.

Data preprocessing and annotation, including peak extraction, baseline adjustment, deconvolution, alignment, and integration, were performed with Chroma TOF software (V 4.3X, LECO), and metabolite identification by matching the mass spectrum and retention index was performed using the LECO-Fiehn Rtx5 database. Finally, the peaks detected in less than half of the QC samples or RSD >30% in QC samples were removed.

### 2.14. Statistical Analysis

Data are represented as mean ± standard deviation (*x* *±* *s*), and they were analyzed using SPSS software (version 19.0). All experiments were repeated thrice, and the results were tested using the homogeneity of variance. Comparisons between groups were tested using a one-way analysis of variance. Statistical significance was set at *P* < 0.05.

## 3. Results

### 3.1. Macroscopic Evaluation Results of Traditional Chinese Medicine

**Figure 2:**
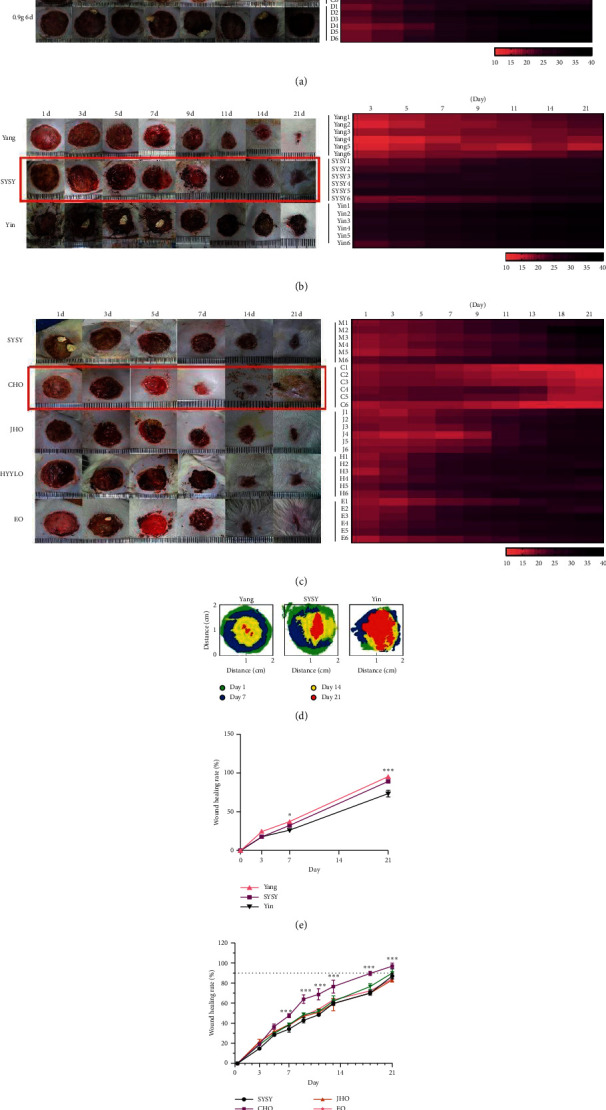
Wounds on the back of rats. (a) Optimal dose and time at which a diabetic ulcer animal model with SYSY syndrome developed. The figure on the left shows wound healing at 8 days after oral administration of different doses of senna leaf. The figure on the right is the heat map of the changes of TCM syndromes of each rat after scoring according to the TCM syndromes rating scale of rats (Yang syndrome: pink < 15; SYSY syndrome: 15 < purple ≤ 30; Yin syndrome: black > 30). (b) Wound healing with different TCM syndromes. The figure on the left shows the wound healing at different time points after the successful establishment of animal models of TCM syndromes. The figure on the right is a heat map of the changes of TCM syndromes over time after the successful establishment of animal models of TCM syndromes. (c) Wound healing of different prescriptions corresponding to different TCM syndromes for treatment of SYSY. (d–f) Wound healing rate. ^*∗∗*^*P* < 0.05, ^*∗*^*P* < 0.01, and ^*∗∗*^*P* < 0.001.

The heat map of rats orally administered with 0.6 g/d for three days was presented in pink, representing Yang syndrome. The heat map of rats orally administered with 0.6 g/d for six days was presented in dark purple during the first four days, representing SYSY syndrome. However, on termination of intragastric administration, the heat map turned red, representing Yang syndrome. The heat map of rats orally administered with 0.9 g/d for three days was presented in purple, representing SYSY syndrome. The heat map of rats orally administered with 0.9 g/d for six days became black on the 8th day, and Yin syndrome was revealed after eight days (Figure 2(a)).

In the Yang syndrome group, red granulation of the wound tissues, yellow and thick pus, shiny white fur, overeating, nonsticky excrement, and high activity were observed. In the SYSY syndrome group, dusky red granulation of wound tissues, less pus, dull and yellow fur, cold tail, sticky excrement, and low activity were observed. In the Yin syndrome group, dark purple granulation of wound tissues, no pus or watery blood, dull and yellow fur, loss of fur, lethargically curling up, and loss of appetite were observed. Pink, purple, and black colors denote the heat maps of Yang syndrome, SYSY syndrome, and Yin syndrome groups, respectively, consistent with the characteristics of differentiation of TCM syndromes of diabetic ulcers (Figure 2(b)). No statistically significant difference was observed in the wound healing rate between the Yang syndrome and SYSY syndrome groups, but a significant difference was observed in the healing rate between Yin syndrome and SYSY syndrome groups (*P* < 0.01; Figure 2(e)).

The heat map of the CHO group changed from purple to pink, indicating that SYSY syndrome gradually developed into Yang syndrome. The color of the heat map in the JHO group changed from purple to light purple and then black, indicating the transformation of SYSY syndrome into Yin syndrome. The color of the heat map of the HYLLO group changed from light purple to dark purple, indicating that SYSY syndrome was transformed into Yin syndrome. The color of the heat map in the EO and JHO groups changed in a similar manner (Figure 2(c)). The wound healing rate was higher in the CHO group than in the other groups, and the difference was statistically significant as compared to that in the SYSY group (*P* < 0.01) (Figure 2(f)).

### 3.2. H&E Staining

**Figure 3:**
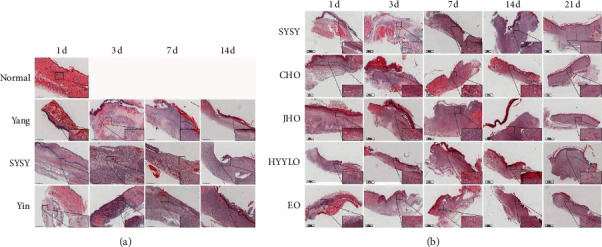
Hematoxylin and Eosin (H&E). (a) The wound healing of rat models with different TCM syndromes at different time points. (b) The wound healing at different time points on the back of rats with SYSY syndrome treated by different TCM prescriptions corresponding to different TCM syndromes (high power image ×400, scale 60 *μ*m; low-power view ×20, scale 600 *μ*m).

On the third day, compared to the Yang syndrome group, a less severe inflammatory cell infiltration was observed in the SYSY syndrome group (Figure 3). On the seventh day, fewer blood vessels were found in the SYSY syndrome group than in the Yang syndrome group. However, in the CHO group, epidermal growth, reduced inflammatory cell infiltration, deposited collagen, and a large number of blood vessels growing perpendicular to the wound were observed. On the fourteenth day, in the SYSY syndrome group, the epidermis grew but could not cover the entire wound surface. Inflammatory cell infiltration was reduced, but still more severe than that in the Yang syndrome group. In the CHO group, the epidermis grew well, and the collagen fibers were orderly arranged towards the epidermis. On the 21st day, the wound surface of the CHO group was completely epithelialized and skin appendages began to grow as compared to that of the other groups, with the least epithelialization in the JHO group.

### 3.3. Masson's Trichrome Stain

**Figure 4:**
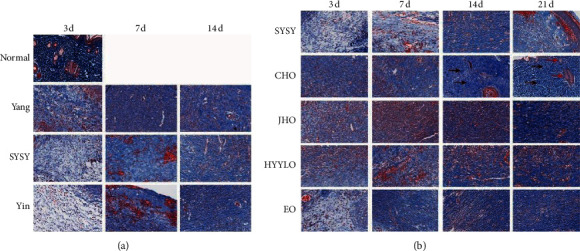
Masson's trichrome staining. (a) The wound healing of rat models with different TCM syndromes at different time points. (b) In the CHO group, black arrows indicate the reticular collagen fibers and the red arrows represent the regenerated sebaceous glands (× 400, scale 60 *μ*m).

The expression of collagen fibers in each group increased with the passage of time. On the third day, the collagen fiber content in the SYSY syndrome group was lower than that in the Yang syndrome group but higher than that in the Yin syndrome group (Figure 4(a)). Compared with other drug treatment groups, the contents of collagen fibers in the CHO group, JHO group, and HYYLO group were similar and were all higher than that in the SYSY syndrome group (Figure 4(b)). On the seventh day, compared with the Yang syndrome group, more free red blood cells could be seen between the collagen fibers in the SYSY syndrome and the Yin syndrome groups. On the 14th day, the content of collagen fibers in the CHO group was the highest, and the fibers were arranged in a dense strip manner. On the 21st day, compared with the other groups, the collagen fibers in the CHO group were arranged in different directions, including transverse, oblique, and longitudinal reticular structures, and regenerated sebaceous glands were visible. Although the content of collagen fibers in the JHO group was high, the fiber bundles fused into films, with the formation of hypertrophic scars (Figure 4(b)).

### 3.4. Antimyeloperoxidase Levels

**Figure 5:**
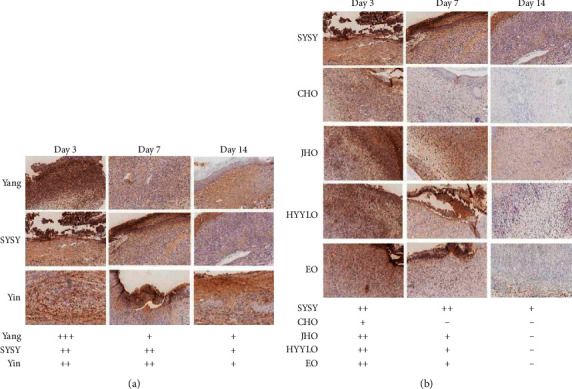
The expression of MPO was detected by immunohistochemistry (×200, scale 200 *μ*m).

On the third day, the MPO expression was highly positive (+++) in the Yang syndrome group, low positive (+) in the CHO group, and positive (++) in the other groups (Figure 5). On the seventh day, the MPO expression in the CHO group was negative (−) in the Yang syndrome group and low positive (+) in the other treatment groups, but still positive (++) in the SYSY syndrome and Yin syndrome groups. On the 14th day, the expression of MPO in each group further decreased. All the treatment groups were negative (−), while the Yang syndrome and the SYSY syndrome groups were both low positive (+).

### 3.5. CD16, CD68, and CD163 Levels

**Figure 6:**
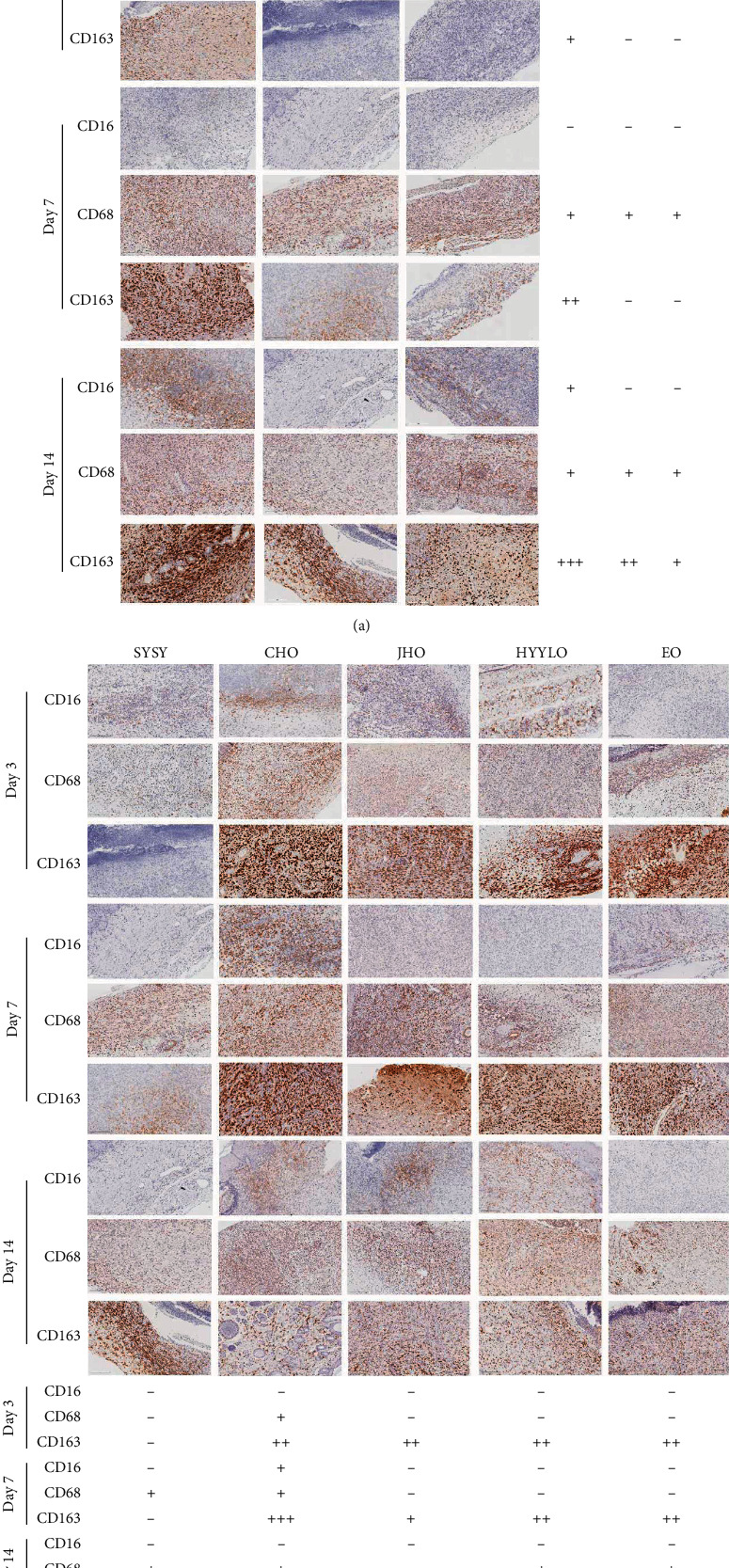
The expression of CD16, CD68, and CD163 was detected by immunohistochemistry (×200, scale 200 *μ*m).

M1 macrophages were marked by CD16 (-), CD68, and CD163 (-), while M2 macrophages were marked by CD16 (++), CD68, and CD163 [14, 15]. On the third day, the macrophages in the Yang syndrome group were activated and were mainly of M1 type, while the macrophages in the SYSY syndrome group and the Yin syndrome group were not activated. The macrophages in each treatment group were also activated and were mainly of M1 type (Figure 6). On the seventh day, the majority of macrophages in the Yang syndrome group were still of M1 type, while only small amounts of macrophages were of M1 type in the SYSY syndrome and Yin syndrome groups. M2 macrophages were the dominant cells in the CHO group, while M1 macrophages were still the dominant cells in the other treatment groups. On the 14th day, M1 and M2 macrophages both existed in the Yang syndrome group, while M1 macrophages mainly existed in the SYSY syndrome and the Yin syndrome groups. On the 14th day, M1 and M2 macrophages both existed in the Yang syndrome group, while the SYSY syndrome and the Yin syndrome groups mainly contained M1 macrophages. The amounts of M1 and M2 macrophages decreased in all treatment groups (Figure 6).

### 3.6. IL-1*β* and IL-10 Levels

**Figure 7:**
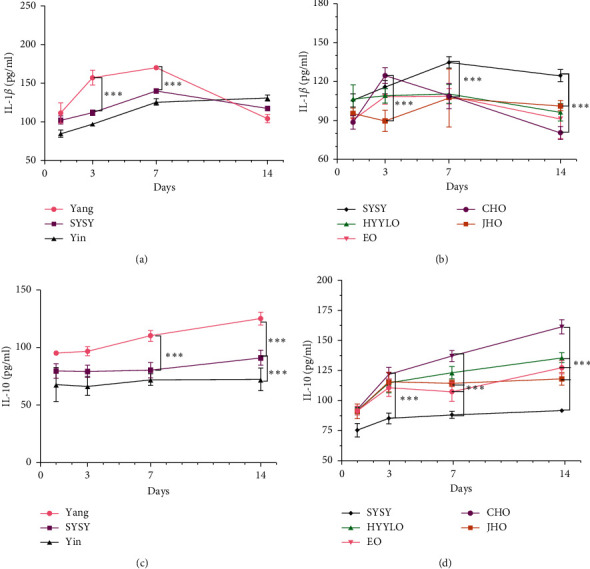
Expression levels of IL-1*β* and IL-10 detected using ELISA. ^*∗*^*P* < 0.05, ^*∗∗*^*P* < 0.01, and ^*∗∗∗*^*P* < 0.001.

On the third day, the IL-1*β* expression level in the SYSY syndrome group was significantly lower than that in the Yang syndrome group (*P* < 0.01) (Figure 7(a)). On the third day, the expression level of IL-1*β* was significantly higher in the CHO group than that in the model group (*P* < 0.01) (Figure 7(b)). On the seventh day, the expression level of IL-10 in the SYSY syndrome group was significantly lower than that that in the Yang syndrome group (*P* < 0.01) (Figure 7(c)), and the expression level of IL-10 in the CHO group was significantly higher than that in the model group (*P* < 0.01) (Figure 7(d)).

### 3.7. CD31 Levels

**Figure 8:**
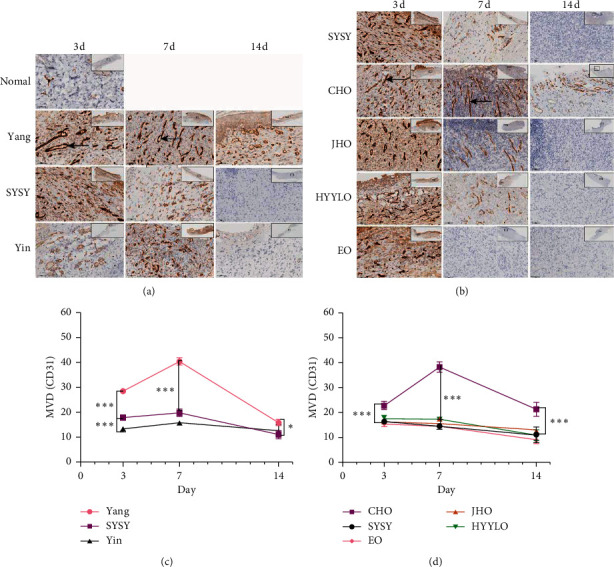
The expression levels of angiogenesis detected using immunohistochemistry (high power image × 400, the ruler is 60 *μ*m; low-power view ×10, scale 800 *μ*m) ^*∗*^*P* < 0.05, ^*∗∗*^*P* < 0.01, and ^*∗∗∗*^*P* < 0.001.

On the third day, the microvessel density (MVD) of the SYSY syndrome group was lower than that of the Yang syndrome group and higher than that of the Yin syndrome group (*P* < 0.01) (Figures 8(a) and 8(d)). CD31 level was higher in the CHO group than in the SYSY syndrome group and other drug treatment groups (*P* < 0.01) (Figures 8(b) and 8(e)).

On the seventh day, most of the capillaries in the Yang syndrome group were trimmed into those growing vertically to the wound surface while the capillaries in the SYSY syndrome group were still immature, disordered, and sparse. Capillaries in the CHO group were slender and orderly, growing perpendicular to the wound surface. The number of capillaries in the SYSY syndrome group was lower than that in the Yang syndrome group and higher than that in the Yin syndrome group (*P* < 0.01) (Figures 8(a) and 8(d)). The number of capillaries in the CHO group was higher than that in the SYSY syndrome group and other drug treatment groups (*P* < 0.01) (Figures 8(b) and 8(e)). On the 14th day, most of the capillaries in the Yang syndrome group underwent apoptosis and gradually formed mature capillary beds. Differently, the capillaries in the SYSY syndrome groups almost disappeared, and the MVD count in the SYSY syndrome group was significantly lower than that in the Yang syndrome group (*P* < 0.01) (Figures 8(a) and 8(d)). The capillaries of the CHO group gradually matured, and the MVD count was significantly higher than that of the other treatment groups and the SYSY syndrome group (*P* < 0.05) (Figures 8(b) and 8(b)).

### 3.8. Results of GC-TOF-MS

On the third day, 112 differentially expressed metabolites, including 69 upregulated and 43 downregulated metabolites, were detected in the Yang syndrome group as compared to those in the SYSY syndrome group. Compared to the SYSY syndrome group, 56 differentially expressed metabolites, including 14 upregulated and 42 downregulated metabolites, were detected in the Yin syndrome group. Compared to the SYSY syndrome group, 143 differentially expressed metabolites, including 65 upregulated and 78 downregulated metabolites, were detected in the CHO group. On the 7th day, 123 differentially expressed metabolites, including four upregulated and 119 downregulated metabolites, were detected in the Yang syndrome group as compared with that in the SYSY syndrome group. Compared to the SYSY syndrome group, 28 differentially expressed metabolites, including 10 upregulated and 18 downregulated metabolites, were detected in the Yin syndrome group. Compared to the model group, 91 differentially expressed metabolites, including 18 upregulated and 73 downregulated metabolites, were detected in the CHO group.

Screening of the differentially expressed metabolites and metabolic pathways is most closely related to the metabolic remodeling of macrophages through the Kyoto Encyclopedia of Genes and Genomes pathway and network analyses.

On the 3rd day, compared to the Yang syndrome and SYSY syndrome groups, the Yang group showed higher expression of such metabolites as lactic acid, gluconic acid, and glycine-2, which were mapped to the pentose phosphate pathway (PPP), indicating an enhanced metabolism of M1-type macrophages. Compared to the Yin syndrome and SYSY syndrome groups, the Yang group showed lower expression of such metabolites as citrate acid and phosphate, which were mapped to the tricarboxylic acid (TCA) cycle, PPP, and oxidative phosphorylation, indicating that the metabolic pathway of M1 type macrophages was weakened. Compared to the SYSY syndrome group, the CHO group showed higher expression of fumaric acid and glucose-1-phosphate, which were mapped to the PPP and pyruvate metabolic pathways, indicating an enhanced metabolism of M1-type macrophages (Table 2). On the 7th day, compared to the SYSY syndrome group, the Yang syndrome group showed lower expression of phosphate, 6-phosphogluconic acid, citric acid, glucose-6-phosphate, and glutamic acid, which were mapped to the TCA cycle and oxidative phosphorylation pathway, indicating an enhanced metabolism of M2-type macrophages. Compared to the SYSY syndrome group, the Yin syndrome group showed higher expression of glutamine-3, which was mapped to the PPP metabolic pathway, indicating an enhanced metabolism of M1-type macrophages. Compared to the SYSY syndrome group, the CHO group had lower citrate, glutamine, pyruvic acid, glucose-6-phosphate1, and 6-phosphogluconic acid contents and glycolysis, which were mapped to the PPP and oxidative phosphorylation metabolic pathways, indicating an enhanced metabolism of M2-type macrophages (Table 2).

## 4. Discussion

This experiment simulated the main cause of SYSY syndrome in clinical practice, that is, treatment failure or incorrect treatment caused by overuse of “cold” drugs for Yang syndrome [5, 6, 16]. The results showed that the CHO group showed the best therapeutic effects, indicating that this animal model can successfully indicate SYSY syndrome. In the process of wound healing, the activation time of macrophages in the SYSY syndrome group was later, and the main manifestation was of M1 type. The inflammatory cytokine IL-1*β* associated with M1 macrophages was expressed at a low level for a long time, while MPO was expressed at a medium level for a long time. IL-10, an anti-inflammatory factor related to M2 macrophages, was also expressed at a low level, and collagen fiber generation was slow and disordered, with poor angiogenesis. The metabolites in the wound tissue of the SYSY syndrome group were compared with those of the Yang syndrome, the Yin syndrome, and the CHO groups. It was found that the expression of different metabolites related to the metabolic pathway of M1 macrophages in the SYSY syndrome group was lower than that in the Yang syndrome group and the CHO group on the third day, but higher than that in the Yin syndrome group. On the seventh day, the expression of metabolites related to the metabolic pathway of M2 macrophages in the SYSY syndrome group was lower than that in the Yang syndrome group and the CHO group, but higher than that in the Yin syndrome group.

According to Xue Medical Records, swelling or no swelling, pain or no pain, ulcer or no ulcer, redness or no redness, and weak pulse are the symptoms and signs of the wounds of SYSY syndrome [16]. This suggests that the wound symptoms of SYSY syndrome are similar to those of Yang syndrome along with Qi deficiency. Therefore, the symptoms and signs of SYSY mentioned above can be evaluated. Qi in TCM science refers to the vital energy that has defense and immunomodulatory functions.

To investigate the pathological changes in diabetic ulcers from Yang syndrome to SYSY syndrome, we used the H&E staining method to observe the pathological changes in the healing process of diabetic ulcers at different time points. It was observed that, in the early stages of wound healing, although the inflammation of SYSY syndrome was lighter than that of Yang syndrome, the inflammation of the Yang syndrome model subsided earlier while the inflammation period of SYSY syndrome lasted longer. The Yin syndrome group showed the mildest inflammation, but it lasted the longest, even more than 14 days. This may be related to the antibacterial effect of aloe emodin, chrysophanol, and other substances in senna leaves [17, 18]. Therefore, inflammatory reactions in the early SYSY syndrome group were lighter than that in the Yang syndrome group. However, anthraquinone derivatives, the main active ingredient of senna, show a more effective purgative action than other anthraquinones. High doses can damage the immunity of rats, and senna is often used to generate animal models related to “deficiency” syndromes. In the Yin syndrome group, the wound edge was sewn into the rubber ring to prevent the contraction of wound surfaces, which exposed it for a long time and led to hypoinflammatory symptoms. Thus, the wound surfaces and physical conditions of rats worsened over time. By comparing the pathological images of the representative TCM syndromes after treatment of the SYSY syndrome, it was found that, in the CHO group, the infiltration of inflammatory cells was obvious on the third day, but the inflammation subsided on the seventh day, with an increase in collagen fibers, epidermal growth, and angiogenesis. Thus, the pathological features of SYSY syndrome are persistent low-grade inflammatory reactions.

In the acute inflammatory stage of normal wound healing, neutrophils arrive at the wound area within a few hours after injury, producing free radicals to kill and engulf dead bacteria [19]. The number of neutrophils peaks at 1–2 days and then gradually decreases. Thereafter, monocytes enter and differentiate into M1 macrophages, which lead to the phagocytosis of apoptotic neutrophils, pathogens, and debris while secreting a large number of proinflammatory factors, such as IL-1*β*. The number of M1 macrophages peaks for 3–5 days and then gradually declines, reaching a stable level by the 14th day. M2 macrophages gradually increased during the process, and they secrete anti-inflammatory factors, such as IL-10, to inhibit the excessive development of inflammation. At the same time, they also secrete growth factors, such as VEGF, TGF-*β*, PDGF, and EGF to promote the differentiation, proliferation, and migration of endothelial cells, leading to angiogenesis, extracellular matrix (ECM) deposition, reepithelialization, and promotion of wound healing [20, 21]. In the early inflammatory stage of wound healing, when the number of M1 macrophages is small, they cannot phagocyte apoptotic neutrophils, bacterial residues, or pathogenic bacteria in time, leading to bacterial escape. Escaping bacteria stimulate M1 to continue to activate. At the same time, a continuous expression of M1 macrophages results in a sustained release of proinflammatory factors, which makes SYSY syndrome the main cause of chronic low-grade inflammation.

Macrophages are the first line of defense in innate immune responses [22]. They can adapt to the environment and perform different functions through metabolic remodeling in different tissues [23]. In the wound healing process, tissue microenvironment changes are characterized by hypoxia and competition for nutrients by large numbers of infiltrating cells, forcing the immune cells to adapt to the microenvironment by reprogramming their metabolism [24, 25]. Lipopolysaccharides and other substances secreted by pathogens activate M1 macrophages, which use aerobic glycolysis to produce energy and molecules for biosynthesis, thus, increasing the flux through PPP to allow NADPH to produce reactive oxygen species (ROS) [26]. The first disruption of TCA cycle results in the accumulation of citrate, which is a substrate for the synthesis of itaconic acid and fatty acids, including prostaglandins [27]. The anabolism of fatty acids produces NADPH, and NADPH oxidase produces ROS [28]. The second disruption leads to the accumulation of succinic acid, which activates hypoxia-inducible factor-1-induced inflammatory gene expression and increases glycolysis [29]. Argininosuccinic acid diverts to increase the levels of fumaric and malic acids required for citric acid production, thus producing nitric oxide and inhibiting succinate dehydrogenase activity.

As the pathogenic bacteria were phagocytized, inflammatory responses reduced. IL-4 and other substances alternately activate M2 macrophages, which are metabolically characterized by increased fatty acid oxidation and decreased glycolysis activity and flux through PPP. Acetyl-coenzyme A, produced by fatty acid oxidation, enters the TCA cycle for oxidative metabolism. L-Glutamine is used primarily for the synthesis of amino and nucleotide sugars, and it also fuels the TCA cycle. Amino and nucleotide sugars are the raw materials used to form collagen fibers and new vessels [29, 30].

Metabolomic results showed that, compared to the other groups, the M1 macrophage metabolic pathway was dominant in the SYSY syndrome group in the early acute inflammation stage, but the metabolic intensity was weaker than that in the Yang syndrome group. After CHO treatment, the M1 macrophage metabolic pathway was enhanced. When the inflammation subsided, metabolism of the macrophages in the Yang syndrome group changed to the M2-type pathway while the SYSY syndrome group still showed the M1 metabolic pathway, which was weaker than the Yin syndrome group, and CHO treatment reversed the M2 macrophage metabolic pathway. This result is consistent with the results of traditional biological experiments.

## 5. Conclusions

In summary, an animal model of SYSY syndrome was established based on the clinical characteristics of diabetic ulcers in this syndrome. SYSY syndrome presents a persistent pathological state of low inflammation, which may be caused by an insufficient activation of the M1 metabolic pathway in macrophages in the early acute inflammatory stage, resulting in the incomplete clearance of pathogens and debris and continuous stimulation of macrophages to initiate the M1 metabolic pathway. The healing and prognosis of SYSY syndrome are related to the proper stimulation of macrophages to activate the reprogramming mechanism. CD163, CD31, IL-10, and the metabolite citric acid may serve as potential specific markers for the recovery and outcome of SYSY syndrome.

## Figures and Tables

**Table 1:** Quantitative scale for traditional Chinese medicine (TCM) syndromes in diabetic ulcer rats.

Site	Symptoms		1	2	3	4	Weighting coefficient
Local wound	Pus	Amount	Small	Moderate	Large	Huge	0.5
		Color	Clear	Yellowish white	Yellowish cloudy	Cloudy/greenish black	1
		Condition	Thick	Less thick	Clear	Watery	1
		Odor	Fishy	Less stinky	Stinky	Blister	0.5
	Slough	Amount	Small	Moderate	Large	Huge	1
		Cast off	Cast off easily from the skin upon pulling	Attached closely	Reoccurrence after treatment	Dry	0.5
	Granulation tissue	Color	Ruddy, soft, and well-developed	Well-developed	Poorly developed	Small amount and greyish white	1
		Granulation particles	Difference in size	Large in size	Swelling	No	0.5

	Epithelial growth		Large area	Large	Moderate	Small	0.5

Physical condition	Color of fur		White and shiny	White and less shiny	Yellowish white and dull	Pale yellow, dull, and fur loss	0.5
	Food		15 g/200 g ≤ and <30 g	30 g/200 g≤ and <35 g/200 g	35 g/200 g ≤ and <45 g/200 g	<20 g/200 g	0.5
	Water		40 mL/200 g ≤ and <100 mL/200 g	100 mL/200 g≤ and 140 mL/200 g	140 mL/200 g ≤ and <150 mL/200 g	>150 mL/200 g	0.5
	Activity		High	Low	Lethargically curling up	Tired and faint	0.5
	Quality of sleep		Well	Moderate	Poor	Sleepiness	0.5
	Excrement (condition)		Normal	Sticky	Watery	Dry	0.5
	Urine (amount)		Normal	A bit more	More	Less	0.5

*Note*. Yang syndrome ≤ 15; 15 < semi-Yin and semi-Yang (SYSY) syndrome ≤ 30; 30 < Yin syndrome ≤ 40; if the value is closer to 22, it indicates that the symptoms are more in line with those of SYSY syndrome.

**Table 2:** Differential metabolites and metabolic pathways related to macrophage metabolic reprogramming in different strategies.

Contrast		Differential metabolite	Metabolic pathways
Day 3	Yang-SYSY	*Glycine-2, lactic acid, and gluconic acid*	Pentose phosphate pathway (PPP)
	Yin-SYSY	**Citric acid and phosphate**	Tricarboxylic acid (TCA) cycle, PPP, and oxidative phosphorylation
	CHO-SYSY	*Fumaric acid and glucose-1-phosphate*	PPP and pyruvate metabolism

Day 7	Yang-SYSY	**Phosphate, 6-phosphogluconic acid, citric acid, glucose-6-phosphate, and glutamic acid**	TCA cycle and oxidative phosphorylation
	Yin-SYSY	*Glutamine-3*	PPP
	CHO-SYSY	**Glutamine-5, glutamic acid, pyruvic acid, 6-phosphogluconic acid, glucose-6-phosphate 1, and citric acid**	PPP and oxidative phosphorylation

*Note.* Italics represents upregulated and **blue** represents downregulated metabolites.

## Data Availability

The datasets used and/or analyzed during the current study are available from the corresponding author upon reasonable request.

## Abbreviations

STZ: Streptozotocin
GC-TOF-MS: Gas chromatography and time-of-flight mass spectrometry
SYSY: Semi-Yin and semi-Yang
TCM: Traditional Chinese medicine
JHO: Jin-huang Ointment
HYYLO: Hui-yang Yu-long Ointment
CHO: Chong He Ointment
EO: Erythromycin Ointment
MPO: Myeloperoxidase
H&E: Hematoxylin and eosin
ELISA: Enzyme-linked immunosorbent assay
MVD: Microvascular density
PPP: The pentose phosphate pathway
TCA: The tricarboxylic acid cycle
ROS: Reactive oxygen species.
